# Designing More Efficient Preclinical Experiments: A Simulation Study in Chemotherapy-Induced Myelosupression

**DOI:** 10.1093/toxsci/kfv316

**Published:** 2015-12-16

**Authors:** Emma C. Martin, Leon Aarons, James W. T. Yates

**Affiliations:** *Centre for Applied Pharmacokinetic Research, Manchester Pharmacy School, the University of Manchester, M13 9PT, United Kingdom and; ^†^AstraZeneca, Innovative Medicines, Oncology, Modelling and Simulation, Li Ka Shing Centre, Robinson Way, Cambridge CB2 0RE, United Kingdom

**Keywords:** 3Rs, preclinical study design, toxicokinetics, pharmacodynamics

## Abstract

A new more efficient preclinical study design (referred to as a compact design) is proposed that removes the need for satellite animals for the collection of toxicokinetic (TK) data by sampling from the main study animals, taking no more than one sample in 24 h to build up a full profile over the course of the study. The compact design’s performance was tested with a simulation study, using an example of chemotherapy-induced myelosupression in rats. Data sets were simulated from a model based on available data, following both the compact design and a traditional design using satellite animals, with 100 studies being simulated for each. The effect of the compact design on parameter and variance estimates for the TK and neutrophil models were investigated, as well as the potential effect of interoccasion variability (IOV). The compact design performed equally as well as the traditional design, and had little impact on parameter or variation estimates, indicating that it would be a suitable alternative to traditional satellite designs while reducing the number of animals required. When IOV was present but not accounted for during the TK analysis some parameter estimates were biased and interindividual variation and residual errors inflated; this was reduced by allowing for IOV in the analysis. Using the compact design removes the need for a satellite group, reducing the number of animals required, without affecting the ability to model the data. If large IOV is suspected, caution should be exercised to avoid parameter estimation bias, and inflation of variability and residual error.

Currently many preclinical studies rely on a separate group of animals (referred to as satellite animals) in order to characterize the toxicokinetics (TK) of a compound. These animals are treated *per-protocol*, but do not undergo the same pharmacodynamic (PD) assessments as the main study animals. One reason for this is that the taking of additional samples required to estimate the TK can lead to physiological or behavioral differences, which could introduce bias in the PD endpoint (Nedelman *et al.*, 1993; Viberg *et al.*, 2012), impacting the integrity of the study. In the example of neutrophil counts, this could take the form of stress, induced by the taking of extra samples, which can cause a decrease in white blood cell count (Mahl *et al.*, 2000; Zeller *et al.*, 1998).

A new innovative design is proposed (referred to as the compact design) for use in preclinical studies with PD endpoints that span multiple dosing occasions, which is tested with a simulation study of chemotherapy-induced myelosupression in rats. It removes the need for satellite animals by allowing the TK to be characterized from the main study animals. The TK samples are taken over multiple days simultaneously with the PD samples, with the aim of reducing stress. The sampling times following dose of the drug remain the same as in the satellite group, allowing a full TK profile to be built up over the course of the study, but only 1 sample is taken on each of the days that PD sampling was scheduled. Meaning the days on which samples are taken are determined by the requirements of the PD, and the time of the sample on that day is determined by the requirements of the TK. This allows a reduction in the number of animals required, in keeping with both the reduction and refinement of the 3Rs principles (Russell and Burch, 1959), as well as being more cost-effective and quicker to run. The 3Rs principles were developed as a framework for treating animals in research humanely, the first principle, replacement, refers to methods that replace the use of animals in experiments, the second, reduction, aims to minimize the number of animals required and the final principle, refinement, refers to the improvement of animal welfare. The design could be further refined by optimizing the TK sampling times and the PD sampling days to make the design more efficient which may allow even fewer animals to be used and further reduce the sampling burden on each animal (Aarons and Ogungbenro, 2010; Bazzoli *et al.*, 2010).

Further, measurement of TK in all animals allows individual parameter estimates to be used when fitting a PD model, instead of relying on population values. Using population values ignores the interindividual variation (IIV) in the TK model which will inflate the variability estimated in the PD parameters (Zhang *et al.*, 2003). Another previous study comparing sequential methods for fitting PD models found that using individual parameter estimates consistently performed better than using population values only, in terms of bias in parameter estimates (Collins, 2013).

Other similar designs have previously been proposed that take 1 or 2 TK samples per day over the course of a study to build up a profile (Nedelman *et al.*, 1993; van Bree *et al.*, 1994; Viberg *et al.*, 2012) and these have been found to be successful when tested on small samples of animals. When using this approach, precautions must be taken to ensure that the volume being sampled falls within current guidelines, such as the European good practice guide (Diehl *et al.*, 2001) which gives maximum sampling volumes over time, with the volume required being dependent on the assay used.

There are 2 main questions that the simulation study aims to address. The first is whether the new design allows the model parameters to be as well estimated as when using a traditional design, despite the change in sampling schedule and the reduction in the overall number of animals. The second is whether by splitting the TK samples over a number of days, interoccasion variability (IOV) could affect parameter estimation. IOV describes variation which occurs within an individual across different dosing occasions, the sources of which are often unexplained. It has been shown that IOV can impact model parameter estimation when ignored, especially when higher than IIV, but this can be avoided by including IOV in the analysis (Karlsson and Sheiner, 1993). The total amount of variation in the data will be preserved, meaning if IOV is ignored during analysis, this variation will instead be attributed to the IIV or residual error, which will become inflated (Ahn and French, 2010; Karlsson and Sheiner, 1993; Laporte-Simitsidis *et al.*, 2000). Misspecification of the variance structure is particularly important if the model is to be used for future simulations (Holford *et al.*, 2000; Mould and Upton, 2013).

## MATERIALS AND METHODS

### 

#### 

##### Development of neutrophil model

Three data sets of absolute neutrophil counts (ANC) were available for the selection and fitting of a model. In each study the investigational cytotoxic was administered orally to rats at various doses and dose intervals, with follow-up to 21 days. In total, data from 136 rats (104 male, 32 female) was available, with doses ranging from 5 to 200 mg/kg and treatment lasting from 1 to 14 days. One study included 21 satellite animals, some of which were sampled following a single dose, and some following multiple doses. No TK samples were taken in the other 2 studies.

**FIG. 1.**
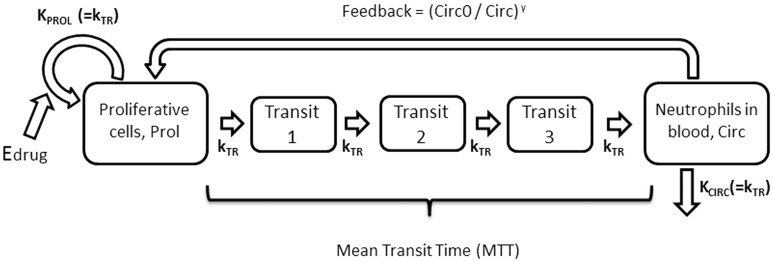
Description of the semiphysiological Friberg model of myelosuppression following chemotherapy, which has been adapted from (Friberg *et al.*, 2002). The k_PROL_ rate parameter describes the production of new proliferative cells, while the k_TR_ rate constant relates to the movement of proliferative cells through transit compartments to the blood where they are observed, k_CIRC_ is then the rate constant describing the removal of neutrophils from the blood. Circ_0_ is the concentration of neutrophils at baseline, and Circ is the observed absolute neutrophil count (ANC), which together describe the feedback mechanism. E_Drug_ is a function of the drug concentration which describes the effect of the drug on the rate at which new neutrophils are created.

The Friberg neutrophil model (Friberg *et al.*, 2002) was chosen to describe the ANC data, which uses both system—and drug-related parameters (Figure 1).

The proliferative compartment represents the proliferative cells such as stem cells and progenitor cells, followed by transit compartments that represent the maturation of cells and finally the circulatory compartment where the count of neutrophils are observed. Circ_0_ is the baseline level of neutrophils, and MTT is the mean transit time (MTT = (n + 1) / k_TR_ where n is the number of transit compartments), which represents the mean maturation time of the neutrophils. E_Drug_ describes the effect of the drug on the neutrophil counts as an inhibition on the proliferation rate. The differential equations are given below (equations 1–5).

(1) $${\text{dProl/dt}=\;\text{k}}_{\text{Prol}}\text{ . Prol}.\left(1-{\text{E}}_{\text{Drug}}\right){\text{ . (Circ}}_{0}{\text{/Circ)}}^{\gamma }-{\text{k}}_{\text{TR}}\text{ . Prol}$$

(2) $${\text{dTransit}1\text{/dt}=\;\text{k}}_{\text{TR}}\text{ . Prol}-{\text{k}}_{\text{TR}}\text{ . Transit}1$$

(3) $${\text{dTransit}2\text{/dt}=\;\text{k}}_{\text{TR}}\text{ . Transit}1-{\text{k}}_{\text{TR}}\text{ . Transit}2$$

(4) $${\text{dTransit}3\text{/dt}=\;\text{k}}_{\text{TR}}\text{ . Transit}2-{\text{k}}_{\text{TR}}\text{ . Transit}3$$

(5) $${\text{dCirc/dt}=\;\text{k}}_{\text{TR}}\text{ . Transit}3-{\text{k}}_{\text{TR}}\text{ . Circ}$$

Where Prol are the proliferative cells and Circ are the neutrophils circulating in the blood. Prol(0)=Transit1(0)=Transit2(0)=Transit3(0)=Circ(0)=Circ_0_.

Data from all 3 studies were combined for model selection and fitting. The TK and neutrophil models were fitted to the data sequentially due to the high computation times associated with simultaneous analysis (∼5 times longer when fitting this model to this data). Individual TK parameters were used where available to fit the neutrophil model, otherwise population values were used.

The first-order conditional estimation method and subroutine ADVAN 13 in nonlinear mixed-effects (NLME) modeling software NONMEM version 7.3 (Beal *et al.*, 2009) was used for all analyses. Fixed effects (typical parameter values) and random effects (IIV and residual error) were estimated. IIV was assumed log-normally distributed. Both additional and muliplicative residual error were tested. The effect of sex on all parameters was investigated. Linear, log-linear and E_MAX_ models were tested as the function providing the link from the TK to the neutrophil model. The number of transit compartments that best described the data were also investigated. All standard errors were confirmed by bootstrapping using Perl-speaks-NONMEM (PsN) (Lindbom *et al.*, 2004) with 1000 replications. A visual predictive check was carried out to evaluate the performance of the model.

##### Performance of compact design

In order to investigate the performance of the compact design in terms of accurately recovering parameter estimates, data from the 2 different study designs were simulated using the final population parameter values estimated during the modeling process above. The simulated satellite design was based on the original dose-range finding study which included 76 rats in total, with satellite animals (∼2 for every 3 in the main study group) to estimate the TK, with 5 TK samples being taken in the 24 h following the first dose. In the compact design, the days on which samples were taken were chosen to be the same as those in the main study group of the original study (baseline and days 2, 4, 8, 12, and 15), the times postdose were then chosen to be the same as those used in the satellite group (0.5, 2, 4, 8, and 24 h), with only 1 TK sample being taken each day, giving a whole TK profile over the course of the study. This compact design reduces the number of animals needed from 76 to 48. For each of the 2 designs 100 data sets were simulated and the same model was then fitted to each simulated data set. The 100 sets of parameters for each design were then summarized, and compared to the values used in the simulation.

##### Impact of interoccasion variability

Splitting the TK samples over multiple days introduces the potential for IOV to bias the TK parameter estimates and the estimation of IIV and residual error; although an advantage is IOV can be assessed with this design. The IOV could not be estimated in the original data as samples from multiple occasions were only available in a limited number of animals. Instead a similar approach was employed to a previous study which assessed the impact of different combinations of IIV and IOV values (Karlsson and Sheiner, 1993). The IIV was simulated at 2 levels, low (32%) and high (55%), on both clearance and volume parameters. Three levels were simulated for IOV; zero, acting as a control, and the same low and high values as used for IIV. IOV was simulated on the clearance parameters only, as in a previous study using the same neutrophil model (Wallin *et al.*, 2010). The impact of IOV on the neutrophil model was not investigated because the samples were taken on the same days in both designs, so potential IOV would affect both designs equally, unlike for TK where IOV would impact the compact design, but not the traditional design. Furthermore, as doses were given daily in both designs, not split into courses of treatment, occasions would be difficult to define for the neutrophil count. Previous studies have shown that IOV in neutrophil counts is low in comparison to IIV (Hansson et al., 2010), minimizing bias (Karlsson and Sheiner, 1993).

The 6 combinations of variation were each simulated 100 times, incorporating IOV in the model using the method outlined in Karlsson and Sheiner (1993). The original model was then fitted to each of the simulated data sets, assuming no IOV was present. In order to investigate whether the presence of IOV could bias parameter estimates, the estimated parameters for each simulated data set were summarized for each combination of values. To assess whether accurately estimating IOV was possible and whether it could improve parameter estimation, the analysis was repeated with IOV estimated, which could then be compared to the results when IOV was ignored.

## RESULTS

### 

#### Neutrophil Model

A 1-compartment TK model was found to be sufficient to describe the TK profiles. Absorption was described as first-order using absorption rate constant k_A_. Concentration-time profiles showed clear differences between males and females, and sex was found to significantly improve the model when included as a covariate on the volume parameter. Males were used as the reference, and had an average volume of distribution of around 12 l/kg, whereas females were estimated to have a smaller volume of 7 l/kg. The estimated parameter values for the TK model are shown in Table 1.

**TABLE 1.** Population Parameter Estimates of Toxicokinetic and Friberg Models

Parameter (units)	Typical Value	RSE%*^a^*	IIV%	RSE%*^a^*
**PK parameters**				
Clearance (l/h/kg)	0.729	10 (10)	57	19 (35)
Volume (l/kg)	12.3	10 (10)	35	23 (23)
k_A_ (1/h)	5.20	34 (58)	—	—
Sex effect	−0.413	19 (20)	—	—
PK residual error (%)	7.12	14 (15)	—	—
**Neutrophil model parameters**				
Circ_0_ (×10^9^/l)	0.873	5 (6)	38	38 (27)
MTT (h)	54.6	3 (9)	23	77 (52)
Gamma (−)	0.670	2 (10)	—	—
Slope (1/µM)	0.0715	0 (14)	44	141 (158)
Neutrophil residual error (%)	9.37	62 (20)	—	—

*^a^*RSE is the relative standard error calculated from the covariance matrix; bootstrapped relative standard errors are given in parentheses.

The Friberg model was fitted to all data, using a linear linking model (equation 6) which was found to give an adequate fit to the data. Different numbers of transit compartments were investigated and 3 was found to be optimal as in other previous uses of this model (Friberg *et al.*, 2002, 2010). The inclusion of sex in the TK model proved sufficient to explain the observed differences, so it was not included as a covariate in the neutrophil model. IIV was found to significantly improve the model when estimated on Circ_0_, MTT and Slope, the same 3 parameters as in the original paper.

(6) $${\text{E}}_{\text{Drug}}=\text{Concentration*Slope}$$

The neutrophil system parameter estimates broadly agreed with another published rat study (Wallin *et al.*, 2010). The previous study showed a similar MTT, 53 h, to that found here, 55 h. Gamma was previously estimated as 0.15 compared to 0.67 here. The ${\text{Circ}}_{0}$ parameter could not be directly compared, as white blood cell counts were modelled in the earlier study, rather than the ANC used here. Model diagnostics were carried out, and the model was found to fit reasonably well, but did overestimate the effects at very low doses. Good agreement was found between bootstrapped standard errors and those output by NONMEM for most parameters.

**FIG. 2.**
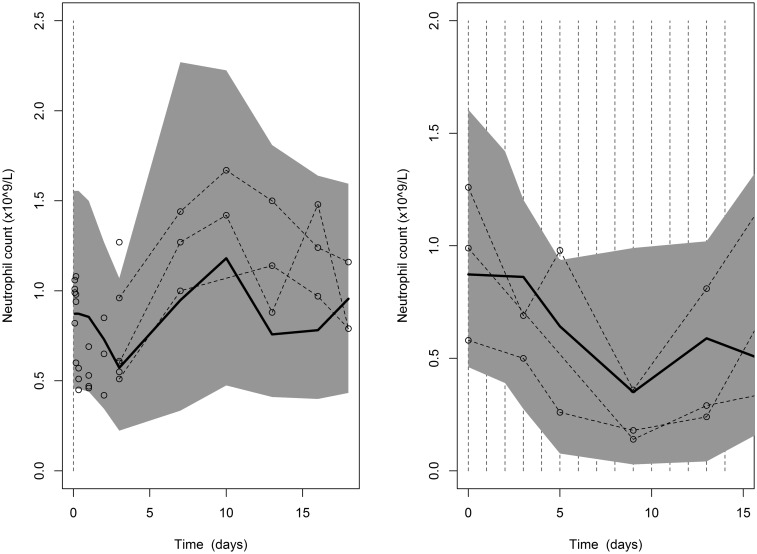
Neutrophil model visual predictive check for 50 mg/kg single dose, with single sample in 18 males, and multiple samples in 3 males (left) and 20 mg/kg 14 daily doses in 3 females (right) with 95% prediction intervals. Vertical dashed lines indicate dosing times.

Examples of the model fit are shown in Figure 2 for two of the dose groups with the most data. When a single larger dose is given (left plot) the neutrophil count quickly drops, reaching the nadir (minimum) around day 4. After day 4, the neutrophil count rebounds, overshoots the baseline and then returns to baseline around day 13. Following the 14 lower multiple doses beginning on day zero (right plot) the nadir is slightly lower, and is not reached until day 9. The count then remains low until around day 16 when follow up ends. The latest sampling time point measured in any animal was 21 days, which does not allow the rebound following 14 daily doses to be fully observed. The 95% prediction intervals have been calculated by simulating 1000 data sets from the model and taking the 2.5th and 97.5th percentiles.

#### Performance of Compact Design

The median parameter estimates from each design are comparable (Table 2), despite the difference in sample size (48 in satellite design compared to 24 in the compact design). In general IIV is well recovered, except in the case of the slope parameter from the linking model, which is overestimated by both designs. Residual error in the neutrophil model is inflated in both designs.

**TABLE 2.** Summary of Parameter Values From 100 Simulated Data Sets Following Satellite and Compact Designs, Median (interquartile range)

Theoretical Values		Satellite Design	Compact Design
**PK parameters**			
Clearance (l/h/kg)	0.729	0.764 (0.722, 0.816)	0.748 (0.694, 0.803)
Volume (l/kg)	12.3	12.0 (11.4, 13.0)	12.1 (11.4, 13.0)
k_A_ (1/h)	5.20	4.65 (4.21, 5.88)	4.73 (4.17, 5.46)
Sex effect (−)	−0.413	−0.392 (−0.459, −0.344)	−0.416 (−0.444, −0.359)
**Neutrophil parameters**			
Circ_0_ (×10^9^/l)	0.873	0.827 (0.795, 0.874)	0.838 (0.780, 0.877)
MTT (h)	54.6	54.4 (50.8, 57.6)	51.6 (49.7, 54.4)
Gamma (−)	0.670	0.306 (0.280, 0.323)	0.301 (0.272, 0.335)
Slope (1/µM)	0.0715	0.069 (0.060, 0.078)	0.077 (0.066, 0.085)
**IIV (%)**			
Clearance	57	53 (47, 58)	55 (49, 58)
Volume	35	34 (30, 37)	33 (29, 35)
Circ_0_	38	37 (34, 40)	38 (34, 42)
MTT	23	21 (19, 23)	20 (19, 22)
Slope	44	71 (64, 78)	67 (52, 72)
**Residual error**			
PK (%)	7.12	7 (6, 8)	7 (6, 8)
Neutrophil model (%)	9.37	10 (10, 11)	13 (11, 15)

**FIG. 3.**
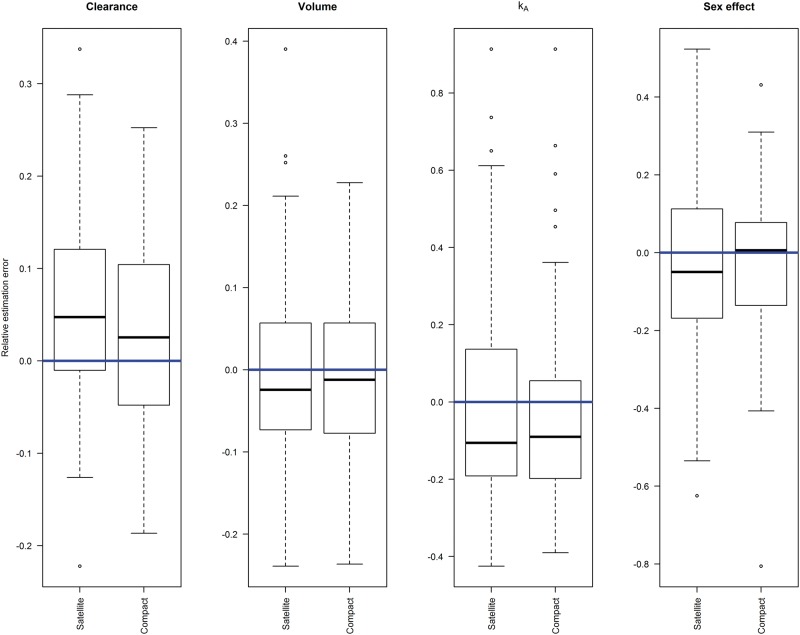
Box plot of relative estimation errors of toxicokinetic parameter values estimated from simulated data sets following satellite and compact study designs, compared with theoretical values (horizontal line), with no interoccasion variability present.

Box plots of the relative estimation errors of the TK parameter estimates for each design (Figure 3) illustrate how the median parameter estimates and variation around them compare to the theoretical value used in the simulation. Collecting the TK data over multiple days (compact design) does not appear to affect the ability to estimate the parameters of the TK model. The shrinkage values for the TK model in the compact design are small, with median of 1% on clearance and 9% on volume, so minimal bias is expected when using individual TK parameter estimates in the neutrophil model (Savic and Karlsson, 2009).

**FIG. 4.**
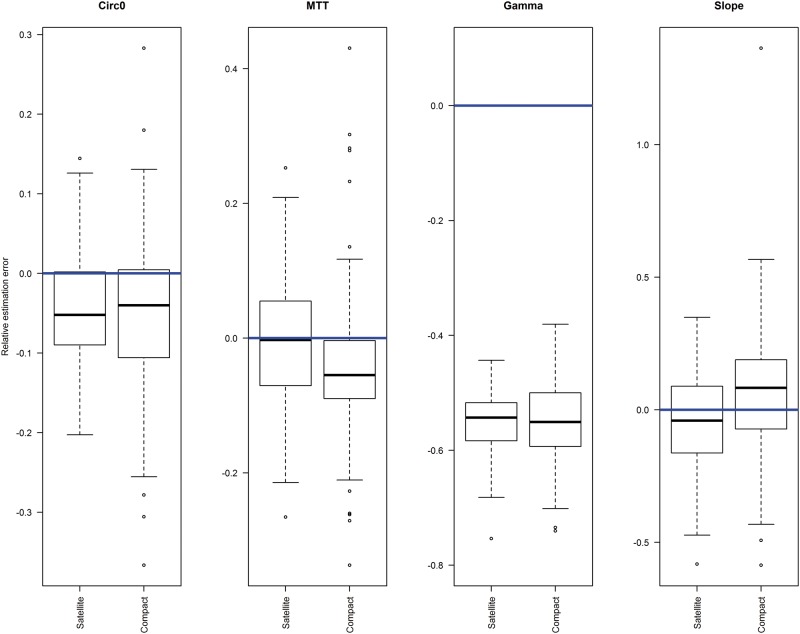
Box plot of relative estimation errors of neutrophil parameter values estimated from simulated data sets following satellite and compact study designs, compared with theoretical values (horizontal line), with no interoccasion variability present.

Similar plots for the parameters of the neutrophil model (Figure 4) also show little difference in the parameter estimates for either design, with Circ_0_ and gamma underestimated by both designs. The variation in parameter estimates appears to be slightly higher in the compact design, which may be a result of the reduction in sample size.

The parameter estimates were close to the theoretical values for both designs; having individual TK data in the compact design did not appear to improve the parameter estimates of the PD model. This could be due to the slow reaction of the model to changing drug concentration. The drug effect is on the rate of change of the neutrophil count, so the resulting changes in neutrophils are therefore dependent on the history of drug exposure over time. This means that misprediction of TK at a particular time is not important. If the predicted drug concentration is correct “on average” over time then the predicted PD will be correct. An alternative explanation is the benefit of using individual TK in the neutrophil model could be being offset by the reduction in sample size.

#### Impact of Interoccasion Variability

When IOV was ignored during analysis, the clearance, volume, and sex effect parameters were well estimated (Table 3), however the estimate of k_A_ became biased, decreasing as the IOV increased. The k_A_ parameter may have been more susceptible to bias as it is the most difficult to estimate, due to little information in the data, however it is expected to have little effect on the neutrophil model. Both IIV and residual error showed inflation when IOV was ignored during analysis, by up to 1.5-fold for IIV and over 3-fold for residual error.

**TABLE 3.** Summary of Parameter Values From 100 Simulated Data Sets When Inter Occasion Variability Was Ignored During Analysis, Median (interquartile range)

		Low IIV			High IIV		
Theoretical Values		No IOV	Low IOV	High IOV	No IOV	Low IOV	High IOV
**PK parameters**							
Clearance (l/h/kg)	0.729	0.75 (0.72, 0.77)	0.74 (0.71, 0.76)	0.70 (0.66, 0.74)	0.76 (0.71, 0.80)	0.73 (0.69, 0.77)	0.70 (0.64, 0.74)
Volume (l/kg)	12.3	12.3 (11.6, 12.9)	11.7 (11.2, 12.5)	11.0 (10.3, 12.0)	12.4 (11.2, 13.4)	11.8 (10.9, 12.9)	11.4 (10.3, 13.1)
k_A_ (1/h)	5.20	4.7 (4.2, 5.9)	3.9 (3.4, 4.4)	2.8 (2.3, 3.6)	5.0 (4.3, 6.2)	3.7 (3.2, 4.3)	3.0 (2.4, 3.5)
Sex effect	−0.413	−0.41 (−0.46, −0.36)	−0.40 (−0.45, −0.36)	−0.40 (−0.47, −0.32)	−0.41 (−0.46, −0.31)	−0.41 (−0.50, −0.34)	−0.42 (−0.51, −0.33)
**IIV (%)**							
Clearance (low or high)	32 or 55	31 (29, 35)	33 (32, 36)	40 (36, 44)	52 (49, 57)	52 (48, 57)	54 (50, 62)
Volume (low or high)	32 or 55	30 (28, 33)	37 (35, 42)	47 (39, 52)	52 (48, 59)	57 (52, 62)	64 (58, 70)
**Residual error**							
PK model (%)	7.12	6.9 (6.3, 7.6)	11.8 (10.8, 12.8)	20.5 (19.4, 22.5)	7.1 (6.6, 7.9)	13.8 (12.3, 15.0)	22.2 (20.1, 25.0)

**FIG. 5.**
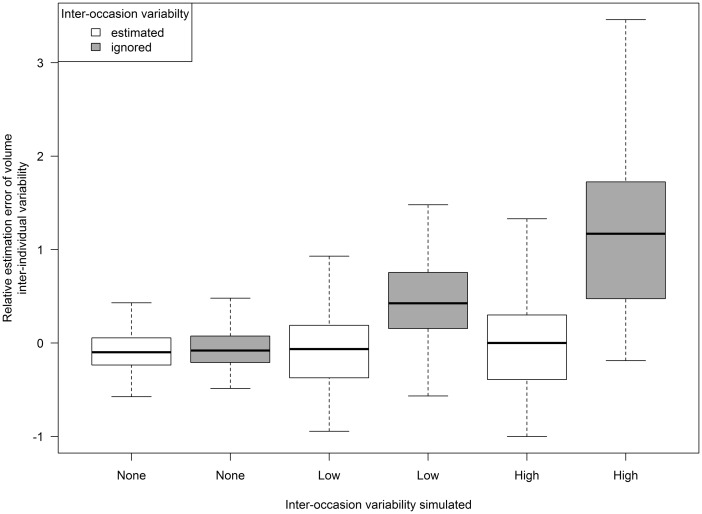
Box plots of estimates of relative estimation errors of IIV on volume parameter to compare interoccasion variability being estimated during analysis (white) and being ignored during analysis (shaded) for increasing levels of interoccasion variability simulated.

When IOV was included in the analysis, the majority of parameters remained well estimated (Table 4), although the k_A_ became inflated when IOV is equal to or higher than IIV, an observation which has been previously reported (Karlsson and Sheiner, 1993). The results show that IOV was well estimated using this design and also improved the estimation of IIV and residual error. An example is shown in Figure 5, where the realtive estimation errors of the IIV on the volume parameter become increasingly inflated as IOV increases when IOV is ignored during analysis. However, this trend is removed when it is estimated. An increase in the variabilty in parameter estimates can be seen as IOV increases regardless of the type of analysis.

**TABLE 4.** Summary of Parameter Values From 100 Simulated Data sets When Inter Occasion Variability Was Included in Analysis, Median (interquartile range)

		Low IIV			High IIV		
Theoretical values		No IOV	Low IOV	High IOV	No IOV	Low IOV	High IOV
**PK parameters**							
Clearance (l/h/kg)	0.729	0.75 (0.72, 0.77)	0.77 (0.74, 0.80)	0.76 (0.73, 0.82)	0.76 (0.71, 0.80)	0.77 (0.72, 0.81)	0.77 (0.71, 0.83)
Volume (l/kg)	12.3	12.4 (11.6, 13.0)	12.5 (11.7, 13.2)	12.6 (11.7, 13.4)	12.4 (11.2, 13.4)	12.4 (11.5, 14.0)	12.9 (11.5, 14.5)
k_A_ (1/h)	5.20	4.9 (4.3, 6.1)	5.4 (4.2, 6.8)	6.2 (4.4, 10.0)	5.2 (4.3, 6.3)	5.1 (4.3, 6.4)	6.6 (4.6, 10.0)
Sex effect	−0.413	−0.40 (−0.46, −0.36)	−0.41 (−0.45, −0.36)	−0.40 (−0.46, −0.34)	−0.41 (−0.46, −0.31)	−0.41 (−0.49, −0.34)	−0.41 (−0.50, −0.32)
**IIV (%)**							
Clearance (low or high)	32 or 55	31 (29, 33)	30 (27, 33)	28 (23, 36)	52 (49, 57)	52 (49, 57)	53 (48, 59)
Volume (low or high)	32 or 55	30 (28, 33)	31 (25, 35)	32 (25, 36)	52 (48, 58)	54 (49, 57)	53 (48, 59)
**IOV (%)**							
Clearance (low or high)	32 or 55	0 (0, 5)	32 (25, 37)	58 (53, 65)	0 (0, 4)	31 (24, 37)	58 (51, 65)
**Residual error**							
PK model (%)	7.12	6.8 (6.1, 7.5)	7.1 (5.3, 8.3)	5.6 (3.2, 7.2)	7.0 (6.5, 7.8)	7.1 (5.2, 8.8)	5.6 (2.2, 9.4)

## DISCUSSION

In these simulation results, a compact design is more efficient than the traditional satellite design, greatly reducing the number of animals required without increasing the number of sampling times, while still achieving the same results. This illustrates 1 way in which NLME modelling can be used, instead of population level estimates, to improve study design, and reduce the number of animals. The compact design still performs well in the presence of IOV, as long as it is accounted for during the analysis, which removes the potential bias in parameter estimates and inflation in IIV and residual error.

Designs similar to the compact design have previously been successful when trialed with small numbers of animals (Nedelman *et al.*, 1993; Viberg *et al.*, 2012). This simulation lends weight to these findings, by testing the design for larger groups of animals and more complex trial designs, with more dose groups and more sampling times, highlighting the many practical advantages and flexibility of these designs.

While the simulation study has only been carried out using 1 model, raising possible questions about generalizability, its success does suggest it could be an alternative design for preclinical studies in other areas. The design could be applied to any study measuring a longer-term PD effect, by selecting the days of samples based on the requirements of the PD and selecting the timings on those days based on the requirements of the TK. The design could be further improved by using optimal design, to select more informative sampling times, possibly allowing for a further reduction in sample size without loss of information.

Utilizing this new compact study design in a preclinical setting would provide numerous advantages over the satellite designs frequently used. The new compact design would allow fewer animals to be used, without additional sampling burden and without impacting on the quality of the data or the breadth of analysis that could be carried out. This makes the compact design substantially more ethical, cost-effective, and quicker to complete. In order to confirm the potential benefits of this design it should be further tested in a preclinical setting. Due to the nature of the design, it could be tested alongside a design with satellite groups, with no additional sampling times being required, allowing for a direct comparison.

## FUNDING

This work was supported by a Biotechnology and Biological Sciences Research Council (BBSRC) industrial Collaborative Awards in Science and Engineering (CASE) studentship award.
